# Bilateral Maternal Pelvic Kidneys Presenting as a Tumor Previa: Sonographic Diagnosis and Obstetric Management

**DOI:** 10.1155/2015/694245

**Published:** 2015-06-02

**Authors:** Eran Weiner, Karina Haratz, Maya Ram, Zvi Leibovitz

**Affiliations:** ^1^Department of Obstetrics and Gynecology, Edith Wolfson Medical Center, Sackler School of Medicine, Tel Aviv University, 58100 Tel Aviv, Israel; ^2^Department of Obstetrics and Gynecology, Lis Maternity Hospital, Tel Aviv Sourasky Medical Center, Sackler School of Medicine, Tel Aviv University, 58100 Tel Aviv, Israel

## Abstract

Renal ectopia occurs when the kidney fails to ascend normally to the retroperitoneal renal fossa. Bilateral cases have also been reported but are very rare. Pregnancy and labor with maternal renal ectopia provides a unique challenge to the obstetricians attempting to prevent damage to the kidneys during labor and allow safe delivery. We describe a case of congenital bilateral pelvic kidneys assessed and diagnosed by 3D sonography as “tumor previa” and managed accordingly.

## 1. Introduction

Renal ectopia occurs when the kidney fails to normally ascend to the retroperitoneal renal fossa (level of the L2 vertebra). The ectopic kidney fails to normally rotate resulting in a shift of the renal axis so that the renal pelvis is directed anteriorly rather than medially.

Ectopic kidneys that fail to ascend above the pelvic brim are commonly called pelvic kidneys. Bilateral cases have also been reported but are very rare [1].

The incidence of renal ectopia is reported as 1 in 1000 autopsies. A similar rate was reported in a study of 13,705 fetuses with antenatal ultrasound examinations performed in a tertiary center in Turkey [2]. Sheih et al. among 132,686 schoolchildren found a lower incidence of 1 in 5000 children in Taiwan [3].

The majority of patients with renal ectopia are asymptomatic. The diagnosis is often made incidentally during routine antenatal or postnatal abdominal ultrasound examinations [4]. In the symptomatic patients diagnosed with renal ectopia the findings at presentation are generally related to urinary tract complications, such as infection, obstruction, and renal calculi. Pregnancy and labor with maternal renal ectopia provides a unique challenge to the obstetricians. The literature concerning the impact of the abnormally located kidneys on the course of pregnancy and labor is both dull and old and did not find any maternal or fetal complications related to renal ectopia.

## 2. Case Presentation

We describe a case of a 29-year-old lady who was referred to our hospital at the 40th gestational week of her first spontaneous pregnancy for counseling regarding an elective cesarean section due to a pelvic mass presenting as a tumor previa.

She was aware of an abdominal ultrasound examination performed at her childhood in which bilateral pelvic kidneys were diagnosed. Her renal function tests were always within normal limits.

**Figure 1:**
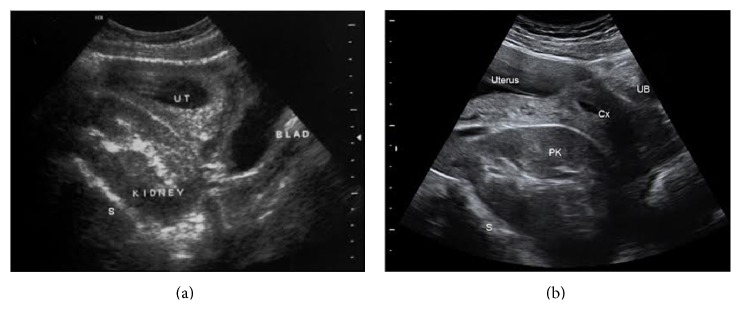
Transabdominal sonograms in the first and second trimesters. (a) shows a sagittal B-mode sonogram performed at the 12th gestational week. The pelvic kidney (PK) lies on the anterior sacral wall (S) below the uterus (UT) containing gestational sac. The inferoposterior wall of the urinary bladder (BLAD) touches the lower pole of the pelvic kidney. (b) demonstrates median plane sonogram performed at 23rd gestational week. Similar to (a) the pelvic kidney (PK) is deeply located in the maternal pelvis in front of the sacrum (S) and behind the uterine cervix (Cx). The uterus appears above the pelvic kidney. The empty urinary bladder is indicated by UB.

On the transvaginal ultrasound examination performed at the 12th gestational week an intrauterine singleton pregnancy with a fetus appropriate for gestational age was found. On this scan the maternal left kidney was demonstrated in the pelvis behind the uterus attached to the sacral wall (see Figure 1(a)).

**Figure 2:**
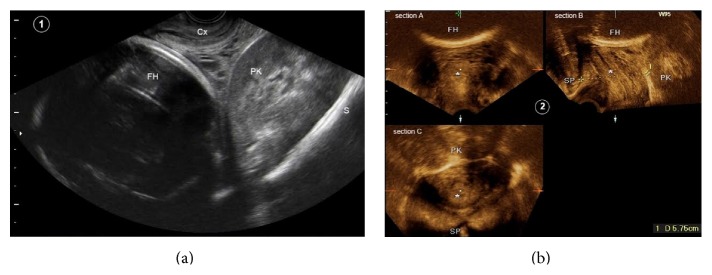
Transvaginal sonograms performed before cesarean section. (a) shows a sagittal B-mode sonogram of the maternal pelvis. The pelvic kidney (PK) is located between the sacral wall (S) and uterine cervix (Cx). The fetal head (FH) is positioned above the kidney and cervix. A three-dimensional multiplanar reformatted sonogram of the maternal pelvis is demonstrated in (b). The 3D volume was obtained by the sagittal acquisition using Voluson E8 ultrasound machine (GE Healthcare) and transvaginal probe (RIC 5-9H/OB). The maternal pelvis is orientated in three orthogonal planes: the coronal plane in section A; the median plane (section B) showing the cervix (*∗*) and fetal head (FH); and the axial plane in section C. Symphysis pubis (SP) and pelvic kidney (PK) appear in the left and right sides of section B, correspondingly. The internal edge of SP and the anterior margin of PK are indicated by the white arrowheads and small white arrows, respectively. The anteroposterior diameter of the pelvic canal between SP and PK was only 5.75 cm. Similarly, in the axial plane (section C) the pelvic canal shows a deformed shape due to the bulging of the pelvic kidney.

Later in the pregnancy, the patient had two routine obstetrical sonographic scans at 15th and 24th gestational weeks. Both examinations were normal, concerning the fetal growth and anatomy. In both scans the bilateral pelvic kidneys were reported (see Figure 1(b)). The kidneys had a normal sonographic appearance and showed no hydronephrosis. The patient was informed of the possibility that the ectopic kidneys may interfere with the vaginal birth and a near-term assessment was recommended. Lastly, she was examined at the 40th week gestation. The fetal heart monitoring and amniotic fluid index were normal and the estimated fetal weight was 3800 grams. On the digital vaginal examination the cervix was closed, 50% effaced, and posteriorly positioned. The fetal vertex was at the spina −5 station, floating outside the pelvic inlet. Below the fetal head, the left pelvic kidney was palpated as a large fixed mass bulging from the upper-posterior vaginal wall into the birth canal. A transvaginal sonogram showing the relation between the fetal vertex, pubis, pelvic walls, and left kidney is presented in Figure 2. An anterior-posterior distance of 5.75 cm was measured between the most anterior margin of the kidney and the posterior aspect of the pubic symphysis, while the fetal biparietal diameter was 9.8 cm.

Based on this information the decision was made to deliver the fetus by an elective cesarean section. At surgery a healthy male newborn weighing 3626 grams was delivered. The right maternal kidney was found in the lower paracolic gutter, and the left kidney was palpated as a retroperitoneal mass behind the lower segment of the gravis uterus. The surgery and postoperative period were uneventful.

## 3. Discussion

Pregnancy and labor with maternal renal ectopia provides a unique challenge to the obstetricians. During pregnancy these kidneys can produce pressure related symptoms such as abdominal pain, backache, and leg swelling [5]. Contrary to expectation that the abnormally located kidney may interfere with the course of pregnancy and labor Anderson [6] and Delson [7] in their separate reviews of renal ectopia in pregnancy did not find supporting evidence concerning maternal and fetal complications or dystocia. The incidence of hypertension was also not significantly raised. However they indicated that the growing uterus may induce pressure effects on the urinary collecting system which might lead to inefficient drainage and provoke urinary tract infection and calculi formation.

In obstetrical and gynecological operations, the pelvic kidney is susceptible to iatrogenic trauma. Kidney contusion or laceration, ureteric devascularization, laceration or ligation, renal arterial occlusion, and renal vein thrombosis could occur and lead to temporary or permanent damage of the renal function.

Invasive procedure, radiological and surgical, may furthermore increase the risk of adhesion formation and infection in renal ectopia, which may, in turn, manifest as urinary tract obstruction, infection, and calculi formation.

Pelvic kidneys in the pregnant patient may behave as other pelvic masses, obstructing the process of labor by forming a “tumor previa.” The most common conditions causing tumor previa are leiomyomas [8] and ovarian tumors [9]. Other rare causes described in the literature are deciduosis peritonei, pelvic hydatid cyst, sacral tumors, cervical carcinoma, and retroperitoneal xanthomatous fibrolipoma. Only one case of a pelvic kidney presenting as a “tumor previa” was previously published. In that case the diagnosis was made early in the labor and an elective cesarean section was performed [10]. We assessed the distance between the most anterior edge of the presacral ectopic kidney and the posterior surface of the symphysis pubis using 2D and 3D sonography. As the measured distance was much shorter than the fetal biparietal diameter a soft tissue dystocia was expected during labor. This accurate sonographic evaluation allowed us to strongly recommend a planned cesarean section in this case. Our cautious management probably enabled safe delivery and avoided possible damage to the ectopic kidney that could have been caused by the pressure of the uterine wall and fetal head during labor.
